# Mucus-Activatable Shiga Toxin Genotype *stx2d* in *Escherichia coli* O157:H7

**DOI:** 10.3201/eid2308.170570

**Published:** 2017-08

**Authors:** Sergio Sánchez, María Teresa Llorente, Laura Herrera-León, Raquel Ramiro, Sandra Nebreda, María Antonia Remacha, Silvia Herrera-León

**Affiliations:** Institute of Health Carlos III, Majadahonda, Spain (S. Sánchez, M.T. Llorente, L. Herrera-León, R. Ramiro, S. Nebreda, S. Herrera-León);; León University Hospital, León, Spain (M.A. Remacha)

**Keywords:** Shiga toxin–producing *Escherichia coli*, STEC, O157:H7, mucus-activatable Shiga toxin, stx2d, whole-genome sequencing, bacteria, Spain, hemolytic-uremic syndrome, zoonoses

## Abstract

We identified the mucus-activatable Shiga toxin genotype *stx2d* in the most common hemolytic uremic syndrome–associated *Escherichia coli* serotype, O157:H7. *stx2d* was detected in a strain isolated from a 2-year-old boy with bloody diarrhea in Spain, and whole-genome sequencing was used to confirm and fully characterize the strain.

The foodborne zoonotic pathogen Shiga toxin (Stx)–producing *Escherichia coli* (STEC) is responsible for human diseases ranging from uncomplicated diarrhea to the life-threatening hemolytic-uremic syndrome (HUS) (*1*). Stx production is the most determining virulence factor implicated in HUS, and the intimin, encoded by *eae*, is the most common adherence factor in HUS-associated STEC (*1*). Stx2d is a Stx2 variant in which cytotoxicity is increased (from 35- to 350-fold) by the action of elastase in intestinal mucus (*2*). This mucus-enhanced toxicity is termed “activation,” and activatable Stx2d proteins are designated Stx2dact. Stx2dact production and Stx2dact genotype (*stx2d*) have been associated primarily with *eae*-negative STEC and considered a predictor for severe clinical outcome in such infections (*3*).

All STEC strains received or isolated in the Reference and Research Laboratory of Food and Waterborne Bacterial Infections (Majadahonda, Spain) are routinely tested for *stx1* and *stx2* subtypes by a PCR subtyping method (*4*). For serotyping, O antigen is identified with both commercial antiserum and PCR (*5*), and H antigen is identified by PCR amplification of the *fliC* gene (*6*) and further sequencing of the PCR product. During 2012–2016, *stx2d* was identified in 7 (3%) of 236 STEC strains isolated from patients with HUS and/or diarrhea in Spain (193 *eae*-positive and 43 *eae*-negative strains). Six were *eae*-negative non-O157 STEC belonging to serotypes O73:H18 (2 strains), O91:H21, O148:H8, O181:H49, and ONT:H21. Strikingly, the other *stx2d*-positive strain identified (CNM-2140/12) belonged to serotype O157:H7 and contained *stx2d* in combination with *stx2c*, apart from *eae*. The strain had been isolated from a 2-year-old boy with bloody diarrhea in July 2012, and it fermented sorbitol after overnight incubation on sorbitol MacConkey agar (Becton Dickinson, Sparks, MD, USA). We confirmed Stx2 production using the enzyme immunoassay kit SHIGA TOXIN QUIK CHEK (TechLab, Blacksburg, VA, USA). The activatable property of the toxin was confirmed by partial sequencing of the *stx2* gene (*4*), analysis of the resulting nucleotide sequence by comparison with the published *stx2* reference sequences, and comparison of the resulting amino acid sequences. The nucleotide sequence of *stx2d* (GenBank accession no. MF094370) was 100% identical to that of strain 06–5231 (O55:H7, GenBank accession no. EF584538). The sequence was translated to amino acids (Technical Appendix Figure), and the activatable property of Stx2d was confirmed with the combined presence of the “activatable tail,” the last 10 aa in the C-terminal end of the A subunit (KSQSLYTTGE), and the END motif at position 14–16 in the N-terminal end of the B subunit (*4*). Stx-associated bacteriophage insertion sites genotyping was performed (*7*). According to the Stx-associated bacteriophage insertion genotype nomenclature proposed by Shringi et al. (*8*), the strain belonged to the SY2c genotype; both *sbcB* and *yehV* loci were occupied, the former with a Stx2c-associated bacteriophage, plus the additional insertion of the Stx2d-associated bacteriophage within the *yecE* locus, as recently reported for *stx2d*-positive STEC O26:H11 (*7*).

To confirm the genotypic traits mentioned earlier, we sequenced the strain using an Illumina NextSeq 500 next-generation sequencer system (Illumina, San Diego, CA, USA). We extracted DNA using a QIAamp DNA Mini Kit (QIAGEN, Hilden, Germany) and generated a genomic DNA paired-end library using a Nextera XT DNA Sample Preparation Kit (Illumina). A total of 1,371,617,258 bp were obtained, providing ≈250-fold coverage and 9,083,558 reads. The sequencing reads have been deposited in the SRA-NCBI public sequence repository (accession no. SRP107062). From the raw whole-genome sequence data, we confirmed serotype O157:H7 using SerotypeFinder; investigated the virulence gene profile, including *stx* subtypes, using VirulenceFinder (Table); and identified acquired antimicrobial resistance genes using ResFinder (all publicly available on the Center for Genomic Epidemiology server, https://cge.cbs.dtu.dk//services/all.php). In addition, we detected mutations in the quinolone resistance–determining regions of the *gyrA* and *parC* genes in silico on the whole-genome sequence, and no acquired antimicrobial resistance gene or mutation conferring quinolone resistance were identified.

**Table:** Virulence gene profile of the *stx2d*-positive Shiga toxin–producing *Escherichia coli* O157:H7 strain CNM-2140/12*

Virulence factor	Identity	Protein function	GenBank accession no.
*ehxA*	100.00	Enterohemolysin	AB011549
*etpD*	100.00	Type II secretion protein	AB011549
*nleA*	100.00	Non-LEE encoded effector A	AE005174
*espP*	99.92	Extracellular serine protease plasmid-encoded	AB011549
*nleB*	99.90	Non-LEE encoded effector B	AE005174
*gad*	98.50	Glutamate decarboxylase	BA000007
*iss*	98.30	Increased serum survival	CP001509
*eae*	100.00	Intimin	AF071034
*iha*	100.00	Adherence protein	AE005174
*tir*	100.00	Translocated intimin receptor protein	EU871626
*stx2B*	100.00	Shiga toxin 2, subunit B, variant c	AB071845
*iss*	98.54	Increased serum survival	CU928160
*astA*	100.00	EAST-1 heat-stable toxin	HM099897
*astA*	91.96	EAST-1 heat-stable toxin	AB042005
*nleC*	100.00	Non-LEE encoded effector C	AP010960
*espA*	100.00	Type III secretions system	AE005174
*gad*	98.50	Glutamate decarboxylase	BA000007
*nleB*	99.90	Non-LEE encoded effector B	AE005174
*espJ*	99.85	Prophage-encoded type III secretion system effector	AE005174
*katP*	100.00	Plasmid-encoded catalase peroxidase	AB011549
*espB*	100.00	Secreted protein B	AE005174
*nleC*	100.00	Non-LEE encoded effector C	AE005174
***stx2B***	**100.00**	**Shiga toxin 2, subunit B, variant d**	**EF584538**

*Determined from raw whole-genome sequence data using VirulenceFinder (https://cge.cbs.dtu.dk//services/all.php). A percent identity threshold of 90% between the input and the best matching database gene was selected. Bold indicates the mucus-activatable Shiga toxin genotype. EAST, enteroaggregative *E. coli* heat-stable enterotoxin; LEE, locus of enterocyte effacement.

Despite its usual association with *eae*-negative STEC, we identified the mucus-activatable Stx genotype also in STEC O157:H7, the most common HUS-associated *eae*-positive STEC serotype. This rare virulence gene combination (*stx2d/eae*) was recently described in STEC O26:H11 isolated from several HUS patients (*9*) and in STEC O80:H2 causing HUS and bacteremia in France (*10*). Although its clinical implications remain unknown—because the strain in our current study was isolated from a patient with bloody diarrhea but not HUS, whereas those isolated in France originated from patients with HUS—these examples show the potential for acquiring and transferring important STEC virulence factors, which can lead to unusual and potentially more virulent strains. Further studies are needed to better determine whether *stx2d*-positive O157:H7 and other *eae*-positive non-O157 STEC strains carrying this *stx* subtype are statistically significant in humans and to determine their clinical implications.

Technical Appendix Alignment with reference strains 06–5231, 5905, C165–02, and B2F1 of the amino acid sequences of the C-terminal end of the A subunit and the N-terminal end of the B subunit of the *stx2d*-positive Shiga toxin–producing *Escherichia coli* O157:H7 strain CNM-2140/12.
