# Supplementary material for: Mucus-Activatable Shiga Toxin Genotype stx2d in Escherichia coli O157:H7
Source: Emerg Infect Dis. 2017 Aug;23(8):1431–3. doi: 10.3201/eid2308.170570 (PMC5547771; doi:10.3201/eid2308.170570)
Supplement: Technical Appendix — Alignment with reference strains 06–5231, 5905, C165–02, and B2F1 of the amino acid sequences of the C-terminal end of the A subunit and the N-terminal end of the B subunit of the stx2d-positive Shiga toxin–producing Escherichia coli O157:H7 strain CNM-2140/12. [file 17-0570-Techapp-s1.pdf]

Article DOI: https://doi.org/10.3201/eid2308.170570

Mucus-Activatable Shiga Toxin Genotype *stx2d* in *Escherichia coli* O157:H7

Technical Appendix

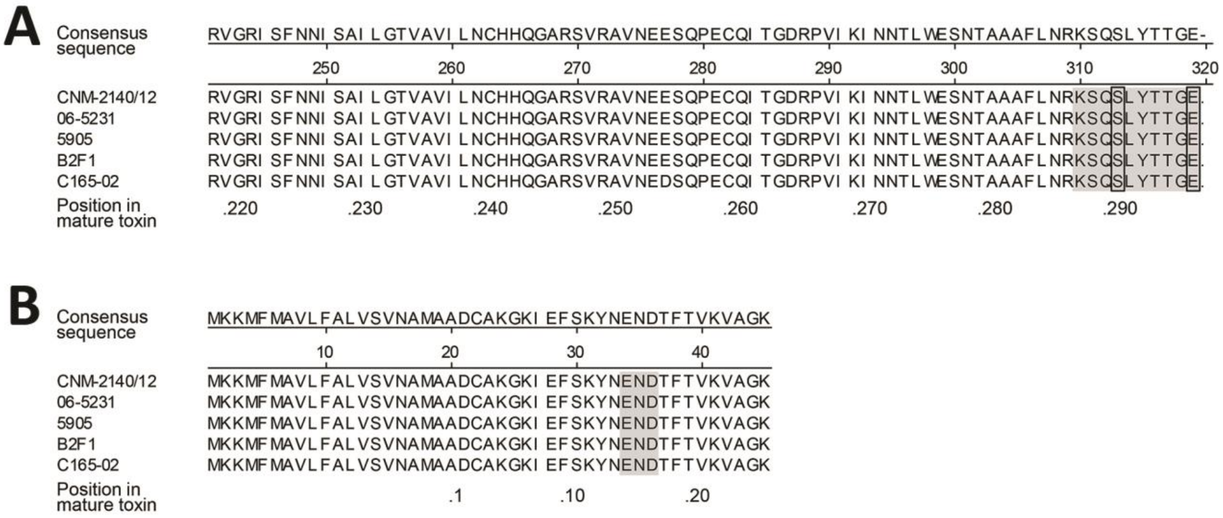

Technical Appendix Figure. Alignment with reference strains 06–5231, 5905, C165–02, and B2F1 of the amino acid sequences of the C-terminal end of the A subunit (A) and the N-terminal end of the B subunit (B) of the *stx2d*-positive Shiga toxin-producing *Escherichia coli* O157:H7 strain CNM-2140/12. Serine (S) at position 291 and glutamic acid (E) at position 297 in the mature toxin defining the activatable tail are included in boxes. Motifs that combined could determine the activatable property of Stx2d are gray shaded.
